# Supplementary material for: Causal Relationship between Aging and Anorexia Nervosa: A White-Matter-Microstructure-Mediated Mendelian Randomization Analysis
Source: Biomedicines. 2024 Aug 16;12(8):1874. doi: 10.3390/biomedicines12081874 (PMC11351342; doi:10.3390/biomedicines12081874)
Supplement: Supplementary file 1 [file biomedicines-12-01874-s001.zip › Supplementary Figure/Figure S4/Singe SNP plot of MR analysis for TL to ubm-b-1596.pdf]

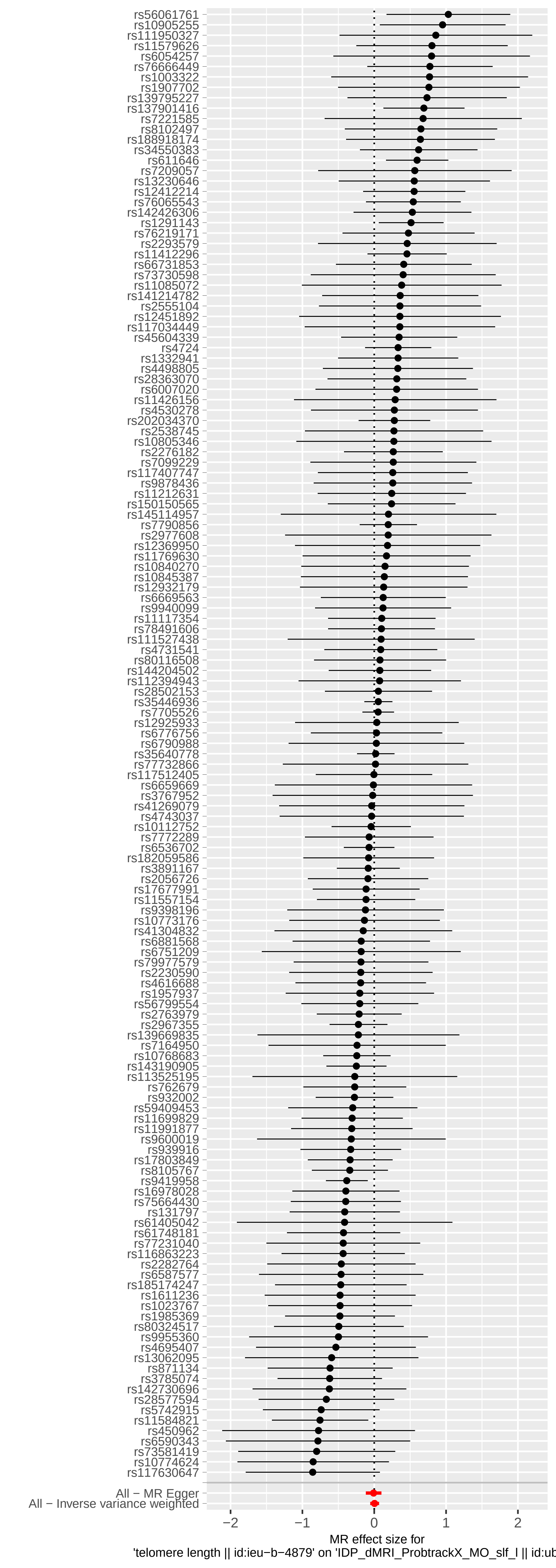
rs56061761
rs10905255
rs111950327
rs11579626
rs6054257
rs76666449
rs1003322
rs1907702
rs139795227
rs137901416
rs7221585
rs8102497
rs188918174
rs34550383
rs611646
rs7209057
rs13230646
rs12412214
rs76065543
rs142426306
rs1291143
rs76219171
rs2293579
rs11412296
rs66731853
rs73730598
rs11085072
rs141214782
rs2555104
rs12451892
rs117034449
rs45604339
rs4724
rs1332941
rs4498805
rs28363070
rs6007020
rs11426156
rs4530278
rs202034370
rs2538745
rs10805346
rs2276182
rs7099229
rs117407747
rs9878436
rs11212631
rs150150565
rs145114957
rs7790856
rs2977608
rs12369950
rs11769630
rs10840270
rs10845387
rs12932179
rs6669563
rs9940099
rs11117354
rs78491606
rs111527438
rs4731541
rs80116508
rs144204502
rs112394943
rs28502153
rs35446936
rs7705526
rs12925933
rs6776756
rs6790988
rs35640778
rs77732866
rs117512405
rs6659669
rs3767952
rs41269079
rs4743037
rs10112752
rs7772289
rs6536702
rs182059586
rs3891167
rs2056726
rs17677991
rs11557154
rs9398196
rs10773176
rs41304832
rs6881568
rs6751209
rs79977579
rs2230590
rs4616688
rs1957937
rs56799554
rs2763979
rs2967355
rs139669835
rs7164950
rs10768683
rs143190905
rs113525195
rs762679
rs932002
rs59409453
rs11699829
rs11991877
rs9600019
rs939916
rs17803849
rs8105767
rs9419958
rs16978028
rs75664430
rs131797
rs61405042
rs61748181
rs77231040
rs116863223
rs2282764
rs6587577
rs185174247
rs1611236
rs1023767
rs1985369
rs80324517
rs9955360
rs4695407
rs13062095
rs871134
rs3785074
rs142730696
rs28577594
rs5742915
rs11584821
rs450962
rs6590343
rs73581419
rs10774624
rs117630647
All − MR Egger
All − Inverse variance weighted
−2
−1
0
1
2
MR effect size for
'telomere length || id:ieu−b−4879' on 'IDP_dMRI_ProbtrackX_MO_slf_l || id:ub
